# SELECTIVE GREY MATTER PATTERNS OF MOBILITY IN OLDER ADULTS WITH AND WITHOUT MULTIPLE SCLEROSIS

**DOI:** 10.1093/geroni/igae098.0148

**Published:** 2024-12-31

**Authors:** Mark Wagshul

**Affiliations:** Albert Einstein College of Medicine, Bronx, New York, United States

## Abstract

Background: Both healthy aging and multiple sclerosis (MS) are associated with changes in cortical grey matter (GM), notably frontal grey matter atrophy consistent with the last in-first out hypothesis, but its relationship to mobility impairments is unknown. We sought to identify brain patterns of cortical GM associated with walking performance, as measured by single- and dual-task walking speed, in older adults with and without MS. Methods: In a cohort of 97 MS and 105 healthy older adults, we measured cortical volume from T1-weighted MRI images and Freesurfer software, along with quantitative gait assessment during natural walking (single-task) and walking while reciting alternate letters of the alphabet (dual-task) conducted within a few weeks of the MRI. Principal components (PC) analysis identified significant latent patterns of GM volume, from the bilateral 68 Freesurfer cortical regions, and pattern scores were entered into regression analyses of gait velocity vs pattern scores, unadjusted and adjusted for demographics variables. Results: The first two PCs showed a significant association with single-task (p = 0.027/0.0025), but only PC2 was associated with dual-task (p=0.082/0.027). In group-stratified analyses, only PC1 was associated with single-task in healthy controls but just below the significance level (p=0.057), and only PC2 was associated with single-task in MS participants (p=0.0052). PC1 consisted of mostly posterior regions (medial occipital, inferior parietal), while PC2 consisted of mostly anterior regions (frontal, cingulate). Conclusion: Grey matter pattern analysis may distinguish between older adults with and without MS, corresponding to anterior, cognitive-control vs posterior, visual/automated control of mobility.

